# Machine learning assisted classification of periodontal health and periodontitis using alveolar bone loss measurements on bitewing radiographs

**DOI:** 10.3389/froh.2026.1814025

**Published:** 2026-05-28

**Authors:** Sunaina Shetty Yadadi, Raghavendra M. Shetty, V. Sowmya, Wael Bakie, Karim Jaziri, Hamza Alsaraierh, Hisham Abu Hijazi, Gadila Siri Reddy, Subhashini Sudhakar, Vineet Vinay, Vijay B. Desai

**Affiliations:** 1 Department of Restorative Dentistry, College of Dental Medicine, University of Sharjah, Sharjah, United Arab Emirates; 2 Microbiota Research Group, Research Institute of Medical and Health Sciences, University of Sharjah, Sharjah, United Arab Emirates; 3 Department of Clinical Sciences, College of Dentistry, Ajman University, Ajman, United Arab Emirates; 4 Department of Pedodontia and Preventive Dentistry, Sharad Pawar Dental College and Hospital, Datta Meghe Institute of Higher Education and Research (Deemed to be University), Wardha, India; 5 Amrita School of Artificial Intelligence, Amrita Vishwa Vidyapeetham, Coimbatore, India; 6 Department of Public Health Dentistry, Sinhgad Dental College and Hospital, Pune, India

**Keywords:** 2018 AAP/EFP world workshop classification, alveolar bone loss, artificial intelligence, automated assessment, bitewing radiographs, machine learning, periodontitis

## Abstract

**Background:**

Periodontitis is a prevalent inflammatory disease characterized by progressive loss of periodontal attachment and alveolar bone. Conventional diagnostic approaches, including periodontal probing and radiographic interpretation, are influenced by examiner variability, limiting consistency in large-scale and community-based assessments. Machine learning-driven models may support standardized screening for periodontal health and periodontitis.

**Aim:**

To develop and validate a machine learning algorithm to classify periodontal health and periodontitis using clinician-measured and validated radiographic alveolar bone loss obtained from bitewing radiographs, aligned with the 2018 American Academy of Periodontology/European Federation of Periodontology (AAP/EFP) classification of periodontal and peri-implant diseases and conditions.

**Methods:**

In this retrospective study, 1,537 of 2,162 bitewing radiographs were included. Alveolar bone loss was measured from the cemento-enamel junction to the alveolar crest using MiPACS software and validated by calibrated examiners (*κ*=0.94). Data were preprocessed, balanced, and split into training, validation, and test sets. Multiple classifiers were benchmarked, with Random Forest (RF) selected after hyperparameter tuning. Performance was assessed using accuracy, sensitivity, specificity, F1 score, and area under the receiver operating characteristic curve (AUC).

**Results:**

The RF model achieved 96.4% accuracy on the validation dataset and 92% on the independent, previously unseen test dataset. Sensitivity was 100% for periodontitis and 93% for healthy cases in the validation set, with an AUC of 0.99. On the unseen test dataset, sensitivity was 94% for healthy and 92% for periodontitis, with an AUC of 0.97.

**Conclusions:**

The machine learning algorithm accurately classified periodontal health and periodontitis from bitewing radiographs, providing an automated assessment aligned with the 2018 AAP/EFP classification. It may improve diagnostic consistency, support early intervention, and enable community-based screening and triage for large-scale periodontal assessment.

**Clinical relevance:**

Automated classification of periodontal status from bitewing radiographs may improve diagnostic consistency and facilitate efficient screening and triage in large-scale and community-based dental care.

## Introduction

Periodontitis is a multifactorial, microbial dental plaque-related, and host-mediated inflammatory disease characterized by degenerative destruction of periodontal attachment (1). Symptoms of periodontitis include bleeding on probing, clinical attachment loss (CAL), formation of periodontal pockets, and radiographically assessed alveolar bone loss, which may lead to potential tooth loss (2). Periodontitis is the sixth most prevalent disease globally and is recognized as the most common chronic inflammatory condition worldwide (3). The global economic cost of productivity loss attributable to severe periodontitis is approximately $54 billion annually (4). The high global prevalence and economic impact of periodontitis necessitate efficient, large-scale screening strategies.

Early detection of periodontal disease remains a challenge, requiring structured preventive strategies to reduce its impact on oral and systemic health. Such initiatives could enhance the individual quality of life while alleviating the economic burden on healthcare systems. Full-mouth periodontal examinations are the “gold standard” but are too time-consuming for large-scale epidemiological studies (5). Radiographic evaluation is commonly used as a supplementary tool to detect periodontal bone loss and offers additional diagnostic information (6).

An automated tool that consistently processes large volumes of radiographic data to identify individuals requiring further examination would mitigate the time-intensive nature of conventional periodontal examination. Artificial intelligence (AI) algorithms can analyze dental images and patient data with greater precision than traditional methods, resulting in earlier and more accurate detection of periodontal disease (7). AI can be an effective aid in reducing dentists' workload, enabling accurate diagnosis and precise, patient-centered care (8, 9).

In the medical field, AI, including machine learning and convolutional neural networks (CNNs) have been applied to detect and diagnose various diseases (10, 11). Some studies have also explored the application of deep learning architectures for detecting periodontal bone loss and early detection of periodontal disease in dentistry (12, 13).

In 2017, the World Workshop standardized the classification of periodontal diseases, introducing a clear framework for periodontal and peri-implant diseases and conditions (14, 15). In 2018, the American Academy of Periodontology (AAP) and European Federation of Periodontology (EFP) adopted the World Workshop on the Classification of Periodontal and Peri-Implant Diseases and Conditions (16). This system incorporates key clinical and radiographic parameters, including probing pocket depth, CAL, and the extent of alveolar bone destruction (14–16). Despite the growing integration of artificial intelligence (AI) in dental diagnostics, only a limited number of studies have aligned AI-driven diagnostic models with the recent classification framework (17, 18). Furthermore, most of these studies have relied predominantly on panoramic radiographs (OPGs) (17–21) and intraoral peri-apical (IOPAs) radiographs (22, 23). AI models trained on panoramic radiographs are constrained by the modality's inherent low image sharpness, superimpositions, and lower resolution (24, 25), which can adversely affect the assessment of periodontitis, particularly for detecting vertical and angular bone defects (26). In contrast, bitewing radiographs offer superior image clarity and diagnostic accuracy for interproximal bone loss (27), which has been proven to be more effective than intraoral periapical radiographs (28). However, bitewing radiographs remain underutilized in AI-based periodontal research.

Recent systematic reviews (29–31) examined AI models for detecting alveolar bone loss due to periodontitis and evaluated their accuracy in classifying periodontal diseases. Evidence from the review, which included numerous studies (17–20, 22, 23), suggested that AI has the potential to aid in the detection of alveolar bone loss and in the classification of periodontal diseases. However, they concluded that further research is needed to standardize AI algorithms and validate their clinical usefulness. Recently, Jundaeng et al. (21) developed a YOLOv8-based deep learning model to diagnose periodontitis by detecting alveolar bone loss on panoramic radiographs and suggested integrating other imaging modalities to improve the efficiency and objectivity of periodontal diagnosis (21). Cassiano et al. (32) developed an AI model using a CNN to identify and quantify periodontal bone loss in bitewing radiographs and recommended that its performance be validated by comparing it with manual measurements by specialists (32).

The precise evaluation of alveolar bone loss is crucial for understanding the severity of periodontitis, as it enables the accurate grading of periodontal disease progression. Moreover, this information is pivotal for developing tailored treatment strategies, whether non-surgical interventions such as scaling and root planing or more advanced surgical procedures. Ultimately, guiding the clinician not only in treatment approaches but also in screening large populations, predicting patient outcomes, and effectively managing long-term periodontal health for better patient care (2, 14, 15, 33, 34).

Hence, the present study aimed to develop and validate a machine learning algorithm to classify periodontal health and periodontitis using clinician-measured and validated radiographic alveolar bone loss from bitewing radiographs, aligned with the 2018 American Academy of Periodontology/European Federation of Periodontology (AAP/EFP) World Workshop classification.

## Methodology

### Study design and ethical considerations

**Figure 1:**
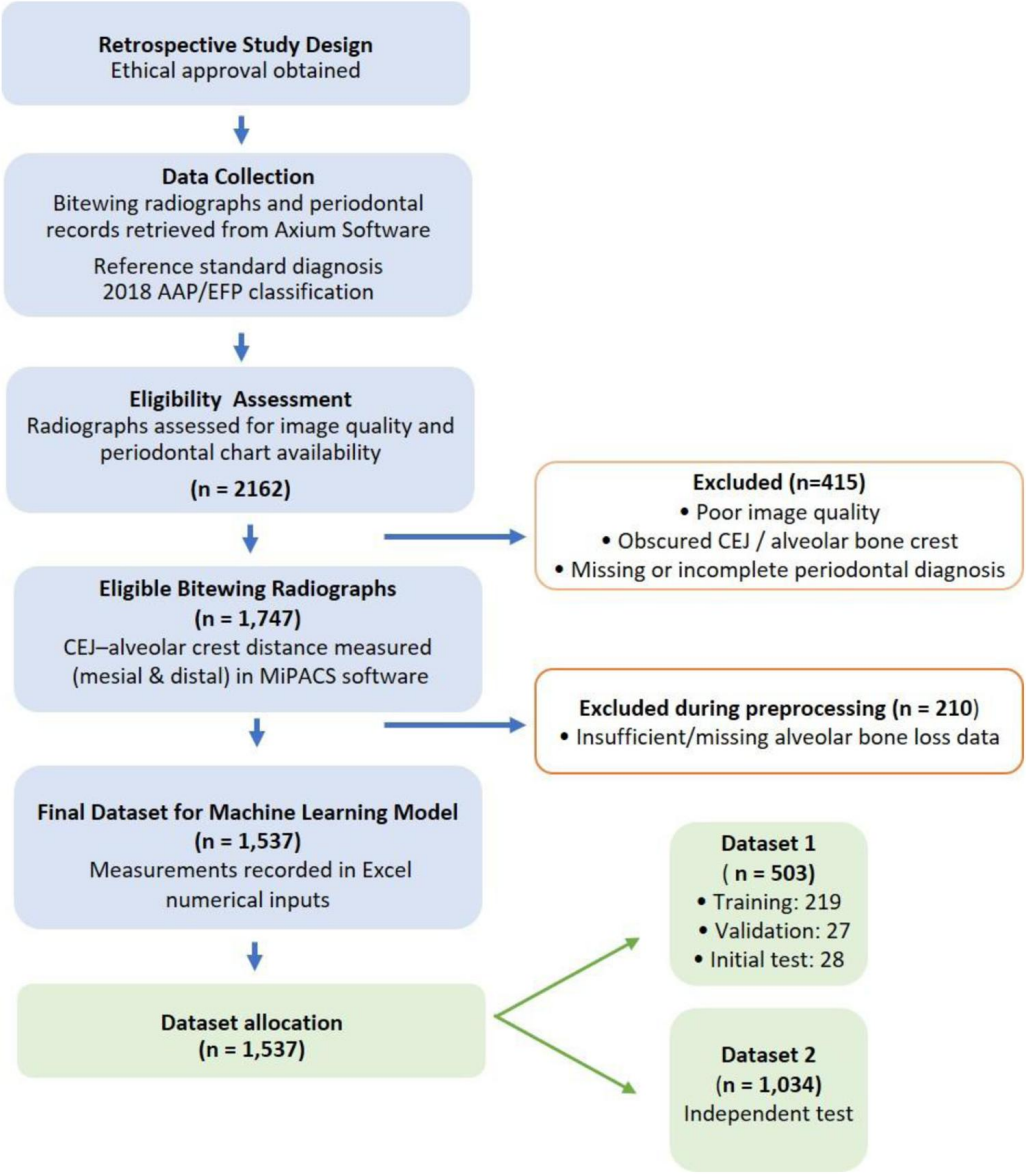
Flowchart of the study.

In this retrospective study, a dataset of bitewing radiographs was used to develop and validate a novel algorithm for classifying periodontal health and periodontitis based on alveolar bone loss measurements. The ‘Research Ethics Committee' of the ‘College of Dental Medicine' at the ‘University of Sharjah' (Ref. No. REC-23-11-25-01-S) approved the study protocol. The reporting of this study adhered to the ‘Transparent Reporting of a multivariable prediction model for Individual Prognosis or Diagnosis - Artificial Intelligence (TRIPOD + AI)’ and the 'Standards for Reporting of Diagnostic Accuracy studies (STARD 2015)’ protocols, ensuring a comprehensive and standardized approach to the methodology and findings (33, 35). The study flowchart is shown in Figure 1.

### Data collection

The bitewing radiographs and periodontitis staging and grading were extracted from the electronic dental record system (Axium; Exam Software, Canada) at the University Dental Hospital, Sharjah (UDHS), Sharjah. The data were collected from patients who attended the Comprehensive Clinic of Restorative Dentistry at UDHS between November 2023 and June 2025. Relevant demographic and clinical information were included to support the assessment of periodontal status. All patient-related data, including bitewing radiographs, periodontal charts, and relevant clinical information, were anonymized prior to analysis. The study adhered to applicable data protection and privacy legislation, including the General Data Protection Regulation (GDPR) and the Health Insurance Portability and Accountability Act (HIPAA).

### Method of staging and grading by dentists and specialists

A team comprising calibrated dental students (WB, KJ, HAH, HA) and two periodontists (SSY and VD) conducted a three-stage data screening process using the Axium software. Examiner calibration was performed under the supervision of the periodontists to ensure consistency in staging and grading.

**Figure 2:**
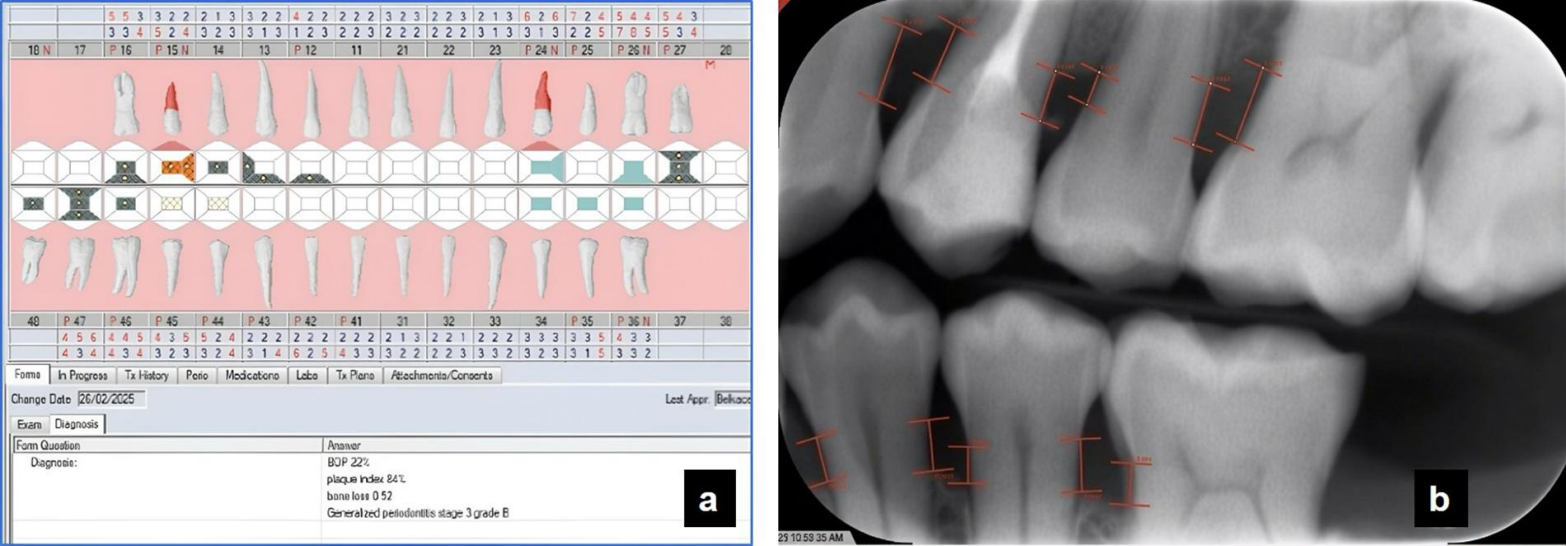
Periodontal Staging and grading done using **(a)** periodontal examination chart from axium software **(b)** radiographs labelled from axium software.

In the first stage, dental students assessed periodontal staging and grading according to the 2018 AAP/EFP classification, using comprehensive periodontal charts (Figure 2a) that included demographic data, risk factors (e.g., smoking, diabetes), CAL, and tooth loss attributable to periodontitis.

In the second stage, bitewing radiographs were evaluated, and alveolar bone loss was measured by determining the distance from the cemento-enamel junction (CEJ) to the alveolar bone crest on mesial and distal surfaces (Figure 2b).

In the third stage, all clinical and radiographic assessments were independently reviewed by two periodontists (SSY and VD). Discrepancies in staging, grading, or bone loss measurements were resolved through consensus, and only cases with concordant clinical and radiographic findings were included as the final reference standard.

### Eligibility criteria

Bitewing radiographs were selected based on image quality and resolution, ensuring clear visualization of the CEJ and alveolar bone crest. Images with obscured or incomplete tooth structures were excluded from the analysis. Additionally, radiographs lacking a corresponding periodontal chart with a definitive diagnosis of periodontitis or health, according to the 2018 AAP/EFP World Workshop classification, were excluded from the study.

### Imaging evaluation and labeling

Among the 2162 bitewing radiographs, a total of 1,747 bitewing radiographs meeting the eligibility criteria were initially included in the study. Using a digital ruler available in the MiPACS imaging software (Medicor Imaging, Charlotte, NC, USA), a line was drawn from the CEJ to the alveolar bone crest for each eligible tooth (mesial and distal surfaces), and bone loss was automatically measured and labeled in millimeters. All examiners were blinded to the machine learning model's predictions during labeling. The index test (machine learning algorithm) used only mesial and distal bone loss measurements derived from the same radiographs. To avoid data leakage, dataset partitioning was performed at the patient level, ensuring that all radiographs from a given patient were included in only one dataset (training, validation, or testing).

The reference standard diagnosis for each patient was “Periodontitis” according to the 2018 AAP/EFP classification, established by consensus based on clinical findings, radiographic data, medical and dental histories, and comprehensive periodontal charting. A patient was definitively classified as “Periodontitis” if the CAL ≥ 3 mm at two or more non-adjacent teeth. This clinical diagnosis required radiographic evidence of alveolar bone loss on bitewing radiographs. Cases with CAL = 0 mm and no radiographic bone loss were classified as “Healthy.” All diagnoses and measurements were confirmed by two periodontists (SSY and VD), with inter-examiner agreement (*κ*=0.94). Further, the discrepancies in diagnosis were resolved through joint review and consensus. Final measurements were recorded in a structured Excel database.

### Sample size estimation and study power

Sample size estimation was based on sensitivity and specificity using the formula:

*n* = (Z^2^ × *P* × (1 – P))/*Δ*^2^, where Z = 1.96 for a 95% confidence level, *P* is the expected sensitivity or specificity, and *Δ* is the margin of error (36). For sensitivity (*P* = 0.96) and specificity (*P* = 0.95), and a target sample size of approximately 1,500, the corresponding margin of error is between 1.1% and 1.2%. These values ensure sufficient statistical power to estimate diagnostic performance with high accuracy and confidence.

## Machine learning methodology

### Training, validation, and testing of the machine learning model

The initial dataset consisted of 1,747 bitewing radiographs. Following preprocessing and data cleaning, 210 radiographs with insufficient alveolar bone loss measurements were excluded, resulting in a final dataset of 1,537 radiographs. This sample size exceeded the minimum requirement, thereby enhancing the robustness of the analysis.

From the final dataset, 503 radiographs were allocated for model development. To address class imbalance, under-sampling was applied, and the data were subsequently split into training, validation, and initial testing sets using an 80:10:10 ratio. After demonstrating strong performance on the initial test set, the remaining 1,034 radiographs were reserved as an independent test dataset for final model evaluation, reflecting a realistic screening scenario. The distribution of samples across datasets is presented in Table 1.

**Table 1:** Data set used for training, validation, and testing of the machine learning model.

Category	Dataset 1	Dataset 1 after under-sampling			Dataset 2
		Train	Validation	Test	
Healthy (Class 0)	137	109	14	14	64
Periodontitis (Class 1)	366	110	13	14	970
Total	503	219	27	28	1034

### Data preprocessing and encoding

**Figure 3:**
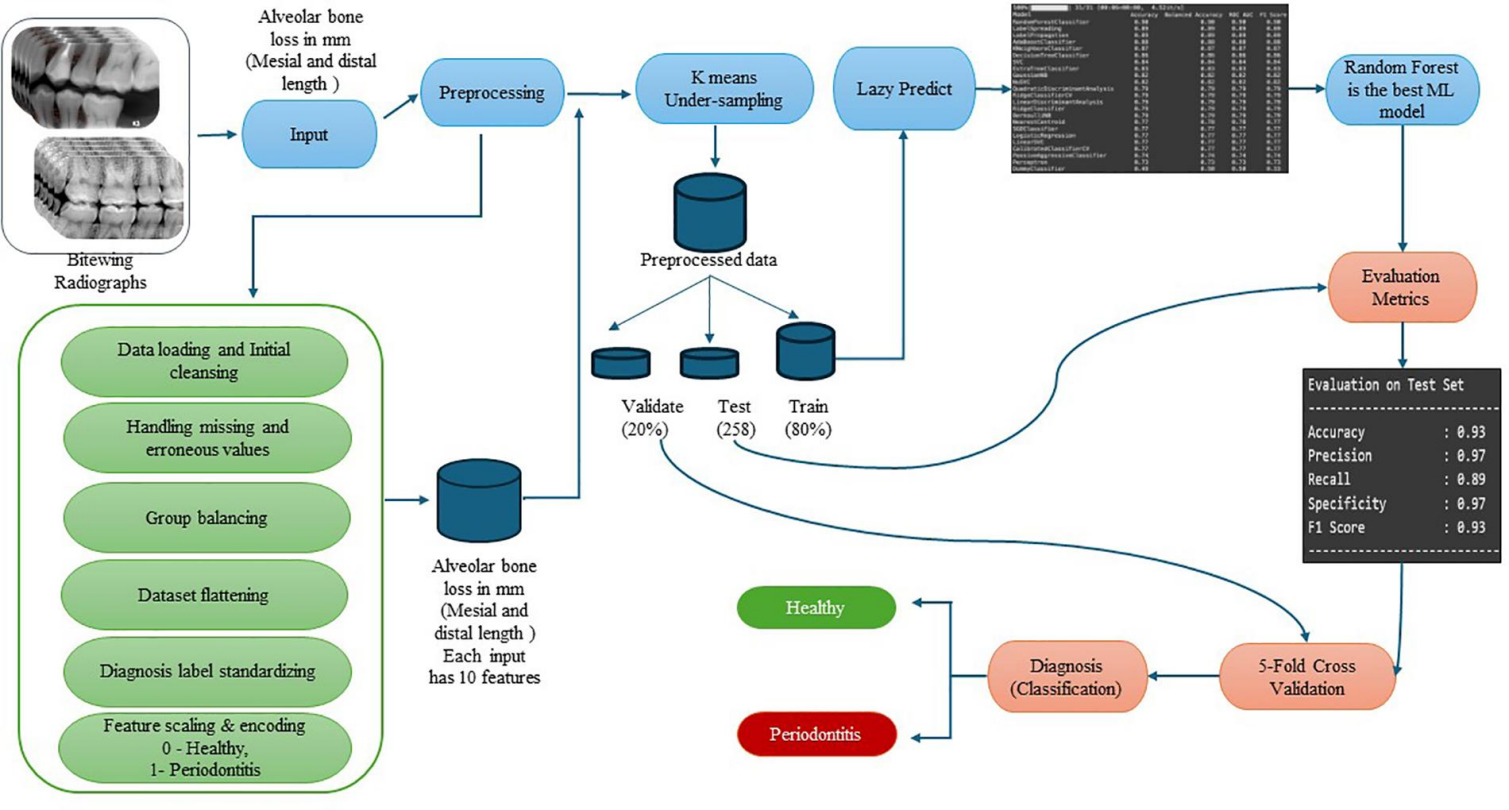
Flowchart of the machine learning model.

The dataset was imported from Excel into a Pandas DataFrame. The dataset was preprocessed in several stages (data loading and initial cleaning, handling missing and erroneous values, group balancing, dataset flattening, group balancing, feature scaling and encoding) to ensure its readiness for analysis (Figure 3). The target variable (‘Patient Diagnosis') was label-encoded, converting categorical values into numerical labels (0 for ‘Healthy' and 1 for ‘Periodontitis'). Feature values were normalized using Min-Max Scaling, bringing all numerical attributes into a comparable range between 0 and 1. The final pre-processed dataset was saved as a CSV file for subsequent model training and evaluation.

### Machine learning models

Machine learning models were developed using mesial and distal alveolar bone loss measurements as input features, with diagnostic labels indicating periodontal status (healthy or periodontitis). Supervised learning algorithms were applied to map these features to diagnostic outcomes and make predictions on unseen data.

### Data balancing and preparation

Due to class imbalance, in which periodontitis cases predominated, test dataset 1 was balanced using K-Means under-sampling. This technique preserved representative samples while reducing the dominance of the majority class by clustering and selecting samples based on proximity to centroids. The balanced dataset was then divided into training (80%), testing (10%), and validation (10%) sets for both classes.

### Model benchmarking

Model benchmarking was performed using the LazyPredict library to enable an unbiased comparison of supervised machine learning classifiers for predicting periodontal status. A total of 24 algorithms were evaluated, including ‘RandomForestClassifier, AdaBoostClassifier, KNeighborsClassifier, DecisionTreeClassifier, SVC, ExtraTreesClassifier, LogisticRegression, GaussianNB, NuSVC, QuadraticDiscriminantAnalysis, LinearDiscriminantAnalysis, RidgeClassifier, RidgeClassifierCV, BernoulliNB, NearestCentroid, SGDClassifier, LinearSVC, CalibratedClassifierCV, PassiveAggressiveClassifier, Perceptron, DummyClassifier, LabelSpreading, and LabelPropagation'.

### Model selection and optimisation

Model selection employed a two-stage approach that combined data-driven screening with domain-informed evaluation. In the first stage, LazyPredict was used to rapidly identify high-performing algorithms based on initial accuracy. Models demonstrating competitive performance were shortlisted for further evaluation.

Among the shortlisted classifiers, Random Forest consistently demonstrated superior performance and stability across validation and test datasets (Table 2). The Random Forest classifier achieved a validation accuracy of 96.2%, with a cross-validation error of 0.01 ± 0.01. Given its robustness, resistance to overfitting through ensemble averaging, and suitability for structured tabular data, Random Forest was selected for final model development.

**Table 2:** Model benchmarking of supervised machine learning classifiers.

Model	Accuracy	Precision	Recall (Sensitivity)	F1 Score
Random Forest	0.96	0.97	0.96	0.96
Label Spreading	0.89	0.89	0.89	0.90
Label Propagation	0.89	0.89	0.89	0.89
Ada Boost Classifier	0.88	0.88	0.88	0.88
K Neighbors Classifier	0.87	0.87	0.87	0.87
Decision Tree Classifier	0.86	0.86	0.86	0.86

To address class imbalance, class weights were adjusted to penalise misclassification of the minority class. Further optimisation using GridSearchCV identified an optimal configuration of 200 estimators with a maximum tree depth of 5, resulting in improved classification performance.

### Feature selection, importance analysis, and model generalization

All input features were clinician-measured mesial and distal alveolar bone loss values from bitewing radiographs, predefined according to the 2018 AAP/EFP classification; hence, no automated dimensionality reduction was used. Feature selection was hypothesis-driven and based on established periodontal diagnostic criteria.

Feature importance was computed using the Random Forest feature_importances_ (Gini-based mean decrease in impurity), normalized to sum to one, allowing comparison of mesial and distal contributions to classification. To reduce overfitting, data were split into training, validation, and independent external test sets. K-Means under-sampling was applied only to the training set. Hyperparameters were tuned via GridSearchCV with cross-validation, and tree depth was limited (max_depth = 5). The ensemble structure further improved robustness.

### Evaluation metrics

The final model was assessed using standard diagnostic performance metrics: accuracy, precision, sensitivity, specificity, and F1 score.

## Results

The model's performance on the balanced test dataset (Dataset 1) is shown in Table 3. Classification accuracy was 96.4%, with high precision and sensitivity for both classes. Weighted averages were calculated to provide a single summary metric, reflecting equal class weighting. All performance metrics are reported with 95% confidence intervals (bootstrapped, 1,000 iterations), demonstrating robust performance under balanced conditions.

**Table 3:** Performance of the machine learning model on the test dataset 1.

Metric	Class 0 - Healthy (95% CI)	Class 1 - Periodontitis (95% CI)	Weighted Average Score (95% CI)
Precision	1.00 (0.85–1.00)	0.93 (0.70–0.99)	0.97 (0.87–1.00)
Sensitivity	0.93 (0.70–0.99)	1.00 (0.85–1.00)	0.96 (0.86–0.99)
F1 Score	0.96 (0.85–0.99)	0.96 (0.85–0.99)	0.96 (0.85–0.99)
Specificity	0.93 (0.70–0.99)	1.00 (0.85–1.00)	0.96 (0.86–0.99)

**Figure 4:**
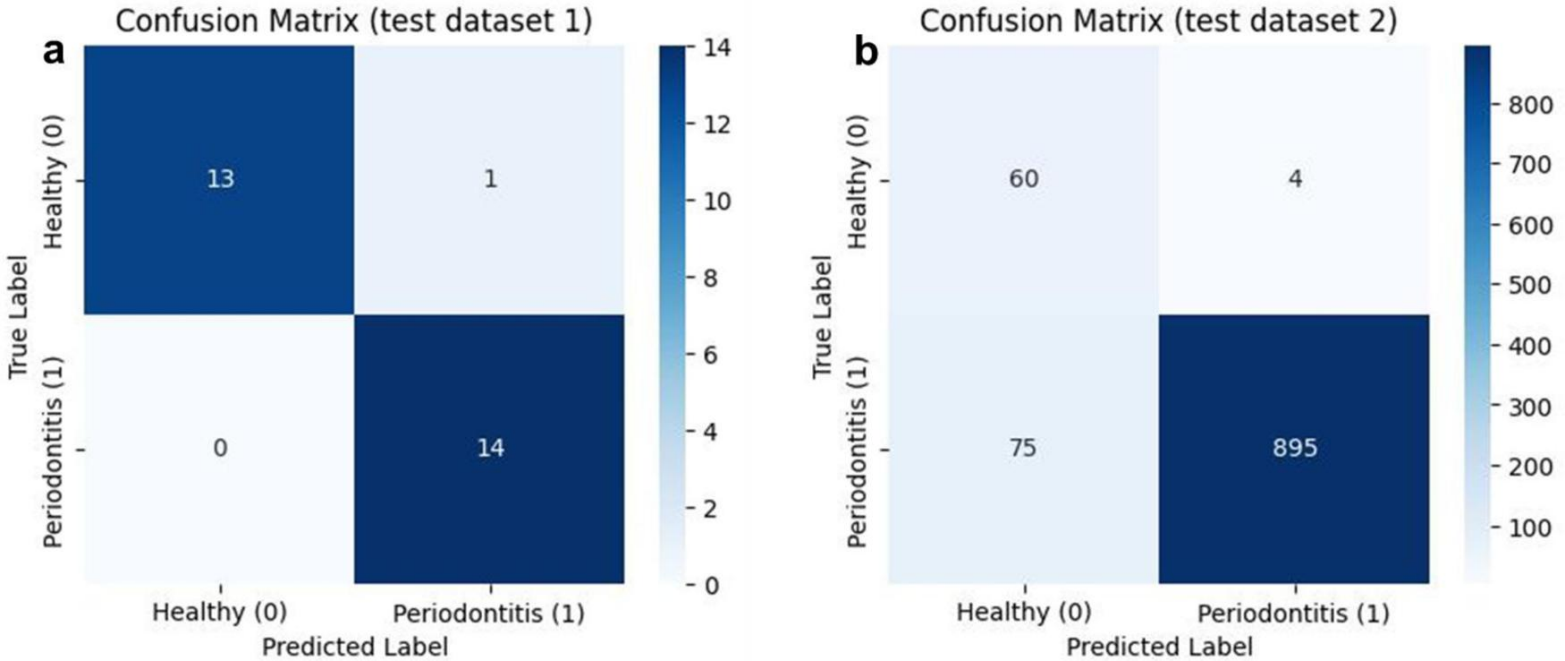
Confusion matrix for **(a)** test dataset 1; **(b)** unseen test data (dataset 2).

The F1 score, which reflects the harmonic mean of precision and sensitivity, is 0.96 for periodontitis and healthy, indicating balanced and robust model performance. The confusion matrix revealed that the model correctly identified 14 periodontitis cases (True Positives) and 13 healthy cases (True Negatives). However, it misclassified one healthy case as periodontitis (False Positives). This discrepancy highlights the model's ability to distinguish periodontitis cases from healthy teeth, as evidenced by the higher sensitivity for the periodontitis class (Figure 4a).

The proposed model was tested on unseen data (1034 bitewing radiographs), which comprise 64 radiographs in Class 0 (healthy) and 970 in Class 1 (periodontitis). The confusion matrix obtained for this test set is given in Figure 4b.

**Figure 5:**
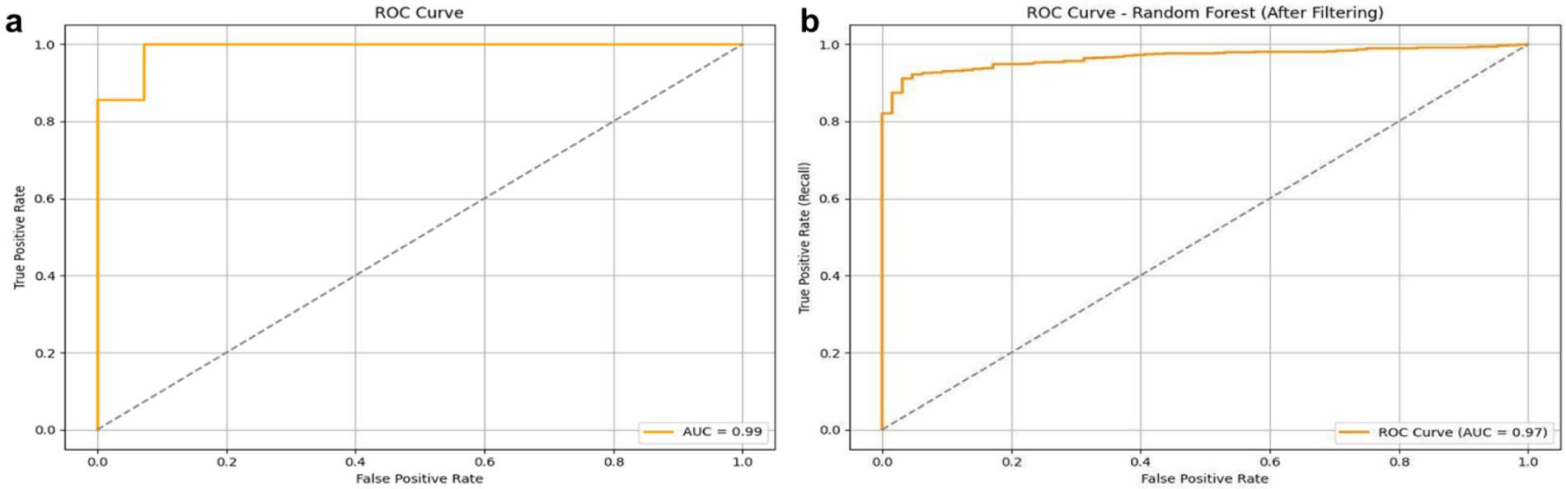
Receiver operating characteristic (ROC) curve for **(a)** test dataset 1;**(b)** unseen test dataset 2.

The Receiver Operating Characteristic (ROC) curve further illustrated the model's classification performance, achieving an Area Under the Curve (AUC) of 0.99, indicating excellent discriminative power. An AUC close to 1 signifies that the model can effectively differentiate between healthy and periodontitis cases. This high AUC is attributed to a comprehensive preprocessing pipeline that included K-Means under-sampling, feature scaling, and the application of a Random Forest classifier with hyperparameter tuning (Figure 5a).

Due to class imbalance in the dataset, performance metrics for the proposed model were evaluated using weighted average scores, as presented in Table 4. The model maintained high sensitivity for both classes (94% for healthy, 92% for periodontitis), though precision for healthy cases was lower (44%), reflecting the model's tendency to prioritize detection of periodontitis. All metrics are reported with 95% confidence intervals derived from bootstrap resampling (1,000 iterations). Furthermore, the model's discriminative performance is supported by ROC, which yielded an AUC of 0.97 (Figure 5b).

**Table 4:** Performance of the machine learning model on the test dataset 2.

Metric	Class 0 - Healthy (95% CI)	Class 1 - Periodontitis (95% CI)	Weighted Average Score (95% CI)
Precision	0.44 (0.33–0.56)	0.99 (0.98–1.00)	0.95 (0.93–0.97)
Sensitivity	0.94 (0.87–0.98)	0.92 (0.90–0.94)	0.91 (0.89–0.93)
F1 Score	0.60 (0.49–0.70)	0.96 (0.94–0.97)	0.92 (0.90–0.94)
Specificity	0.99 (0.97–1.00)	0.44 (0.33–0.56)	0.45 (0.34–0.56)

### Feature importance analysis

**Figure 6:**
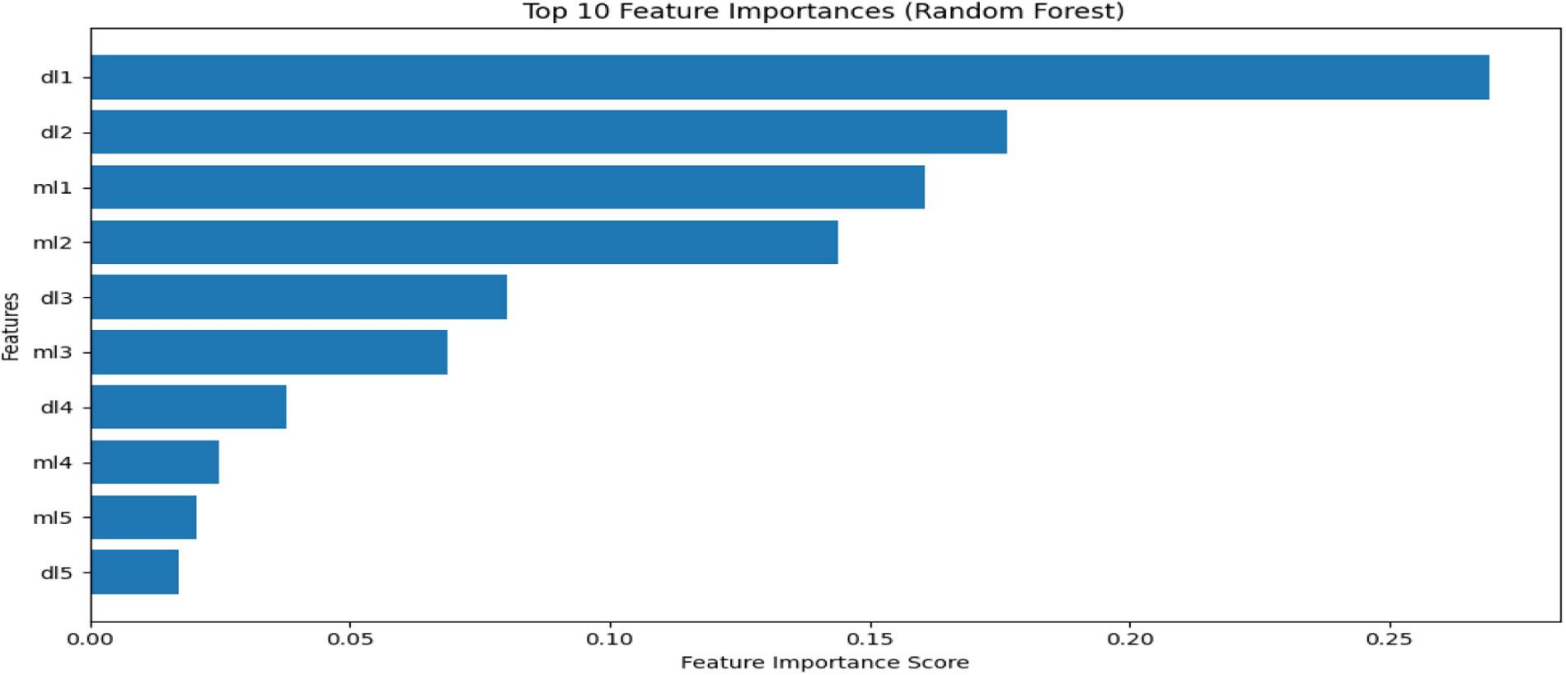
Feature importance analysis.

Feature importance analysis demonstrated that distal measurements, particularly dl1 and dl2, exhibited the highest relative importance scores, followed by mesial measurements ml1 and ml2. These features accounted for the largest proportion of the model's predictive contribution, while higher-index mesial and distal measurements showed progressively lower importance values (Figure 6).

The distribution of importance across multiple related mesial and distal measurements indicates that the model relied on several clinically meaningful interproximal bone loss parameters rather than a single dominant predictor. This distributed contribution pattern reflects stable learning of underlying patterns of bone loss within the dataset, rather than reliance on isolated features.

Importantly, these findings do not imply causal relationships; rather, they indicate that, within the dataset studied, certain mesial and distal regions contributed more strongly to classification performance. The feature-importance ranking supports the clinical relevance of interproximal bone loss measurements for distinguishing periodontal health from periodontitis.

The trained model shows promise in assisting periodontists with rapid diagnosis, particularly when evaluating large datasets, achieving an overall accuracy of 92%. Nevertheless, a reduction in precision for the healthy class and specificity for the periodontitis class was noted.

## Discussion

Identifying periodontal bone loss and accurately classifying periodontal diseases are crucial for an effective periodontal treatment plan. The classification system introduced by the AAP/EFP categorizes periodontal disease using a multidimensional approach that includes staging and grading.16 Staging refers to the severity and extent of disease, whereas grading reflects the rate of disease progression (14–16). Radiographic bone loss (RBL) is a significant factor in determining the severity and progression of periodontal disease. It serves as an objective and dependable method for evaluating periodontal health and categorizing the severity of periodontal disease (37, 38).

The present study advances current periodontal AI research by introducing a transparent, clinician-driven machine learning model based on bitewing radiographic bone loss measurements, aligned with the 2018 AAP/EFP classification, and optimized for screening and triage.

Machine learning has demonstrated the potential to improve diagnostic accuracy and efficiency by minimizing human error (39, 40). By being trained on labeled radiographs, the AI model can distinguish between healthy and diseased conditions and assess the severity of periodontitis (41). The present study aimed to develop and validate a machine learning algorithm to classify periodontal health and periodontitis using clinician-measured, validated alveolar bone loss from bitewing radiographs, aligned with the 2018 American Academy of Periodontology/European Federation of Periodontology (AAP/EFP) World Workshop classification.

The results demonstrated that the machine model achieves high diagnostic accuracy and reliability in detecting periodontal bone loss. The model achieved outstanding performance on the balanced test set (accuracy: 96.42%, AUC: 0.99) and preserved strong, clinically meaningful predictive ability on a large, unseen, and imbalanced dataset (weighted accuracy: 92%, AUC: 0.97), with strength in correctly identifying cases of periodontitis. Beyond its diagnostic accuracy, the primary clinical utility of this model lies in its potential for large-scale screening and triage. In addition, the interpretability of Random Forest through feature-importance analysis supported its suitability for a clinically applicable screening model (42).

The diagnostic performance observed in this study is strongly influenced by the intentional use of bitewing radiographs and a clinician-driven machine learning framework. Compared with panoramic and periapical radiographs, bitewing images provide superior visualization of interproximal alveolar bone levels with minimal distortion, making them particularly suitable for detecting early and moderate periodontal bone loss (24, 28). While recent studies have applied deep learning models to panoramic and bitewing radiographs, these approaches typically rely on raw pixel-level analysis, which limits interpretability and clinical transparency (18, 32). In contrast, the present study employed a traditional machine learning model trained on clinician-measured cemento-enamel junction-to-alveolar bone crest distances, measurements routinely obtained in clinical and educational settings and directly aligned with the 2018 AAP/EFP classification framework (15, 16). Consequently, the model is best positioned not as a definitive diagnostic system but as a high-sensitivity screening and triage tool capable of efficiently identifying individuals with radiographic evidence of periodontal bone loss for further comprehensive periodontal evaluation.

Our findings are highly consistent with those of Tsoromokos et al. (41), who also applied machine learning to predict alveolar bone loss (41). Although their model showed strong performance, it was trained on a relatively small dataset of periapical and panoramic radiographs. The use of a large, high-quality dataset of bitewing radiographs in this study enhances the robustness of the findings. Although cone-beam computed tomography has demonstrated superior diagnostic accuracy for detecting periodontal bone defects (43), its higher cost, increased radiation exposure, and limited accessibility restrict routine clinical use. In contrast, the present model achieves clinically acceptable accuracy using standard 2D bitewing radiographs, supporting its suitability for routine screening and triage. Unlike recent CNN-based approaches that analyze raw image pixels, this study employed a machine-learning model trained on clinician-measured alveolar bone loss, thereby enabling direct integration with established clinical measurement protocols. While deep learning methods may capture more complex image features, future work could explore hybrid approaches combining both strategies.

This study is novel in its systematic application of Lazy Predict and GridSearchCV for model optimization and benchmarking, enabling the identification of the most efficient classifier with transparency and reproducibility. Our approach aligns with that of Patel et al. (44), who highlighted the importance of applying machine learning to large-scale dental datasets for predicting trends in periodontal disease. Through LazyPredict, the Random Forest emerged as the optimal classifier due to its robust performance, resistance to overfitting, and interpretability. Its ensemble architecture, aggregating multiple decision trees, effectively models the complex, multidimensional relationships between mesial and distal bone measurements across teeth while minimizing overfitting.

Although only mesial and distal bone loss measurements were used as predictors, Random Forest models can capture complex non-linear relationships between multiple interproximal measurements across teeth. Furthermore, the machine learning framework allows scalable integration of additional clinical and demographic predictors in future models.

The methodology of this study is closely aligned with the framework established at the 2018 AAP/EFP World Workshop. The diagnostic categories (“Healthy” or “Periodontitis”) assigned to each radiograph were not solely based on radiographic bone loss; rather, they resulted from a thorough, multi-step process that included detailed clinical charting, medical history, and risk factor assessment, all of which were confirmed by a calibrated, multidisciplinary team. This approach ensured that the AI model was trained to make predictions based on a clinically relevant, gold-standard diagnosis. Although the model relies exclusively on radiographic data, its effectiveness is measured against a robust clinical validity. Therefore, this research directly addresses a significant gap highlighted in recent systematic reviews (29, 30), which emphasize the need for AI tools to be consistent with contemporary classification systems and to be validated by expert-led diagnoses.

On the large, imbalanced test set (Dataset 2), the model demonstrated high sensitivity (94% for healthy, 92% for periodontitis) but lower precision for healthy cases (44%) and reduced specificity for periodontitis (44%). This indicates a strong ability to detect true disease cases while occasionally misclassifying healthy individuals as diseased. Misclassified periodontitis cases often had CAL <2 mm, reflecting the inherent difficulty of identifying Stage I disease, which radiographically and clinically resembles health. These findings define the model's optimal role as a screening and triage tool that efficiently identifies clear cases of bone loss while directing borderline cases to expert review, thereby enhancing clinical workflow. Given its high sensitivity, broader deployment of the model may generate false-positive referrals, which should be addressed through standard clinical periodontal examination.

This study was prompted by a recent systematic review conducted by Zhang et al. (31), which highlighted the limited use of bitewing radiographs in AI-driven periodontitis research, despite their superior ability to visualize interproximal bone loss. In alignment with recommendations for clinically integrated, rigorously validated models, our approach follows the 2018 AAP/EFP World Workshop classification, ensuring that all diagnoses are confirmed by a multidisciplinary team of specialists. By developing a robust Random Forest classifier on a meticulously curated dataset of bitewing radiographs, we aimed to address these gaps and translate the algorithm's predictive capabilities into evidence-based clinical practice. In the future, this framework could be integrated with deep learning–based imaging algorithms to enable automated staging and grading of periodontal disease.

Although the model currently performs binary classification, its primary clinical value lies in its application as a high-sensitivity screening and triage tool. By providing automated and reproducible measurements of alveolar bone loss, an essential quantitative parameter within the 2018 AAP/EFP World Workshop classification, the model supports efficient identification of individuals requiring further periodontal evaluation. Automation of this measurement process reduces the burden of manual assessment, enabling clinicians to integrate radiographic findings with additional staging and grading factors, including furcation involvement, tooth mobility, and overall treatment complexity. The use of clinician-derived cemento-enamel junction to alveolar bone crest measurements, rather than raw image pixels, ensures interpretability and direct compatibility with routine clinical and educational workflows.

### Limitations and future prospect

Despite its promising performance, this study has several limitations. The model was developed and internally validated on a retrospective dataset from a single center in Sharjah, UAE, which limits the immediate generalizability of our findings to other populations and geographical settings. The class imbalance in our test set and the model's current binary classification also present constraints. A notable limitation is the absence of demographic and clinical variables due to the use of de-identified retrospective radiographic data. External validation using independent multi-center datasets from diverse institutions and populations is essential to confirm the model's generalizability before clinical deployment.

Future research should prioritize external validation through prospective, multicenter studies involving larger and demographically diverse cohorts. This approach is essential to confirm model robustness, assess predictive performance across varied populations, and support the development of a fully automated system for clinical staging and grading of periodontal disease.

## Conclusions

The machine learning algorithm accurately classified periodontal health and periodontitis from bitewing radiographs, providing automated classification aligned with the 2018 AAP/EFP classification. It represents a proof-of-concept machine- learning framework for automated screening and triage of radiographic bone loss requiring further external validation before clinical deployment.

Although it is currently limited to binary classification, this research lays the groundwork for the future integration of deep learning-based imaging modalities that incorporate risk factors, medical history, and clinical factors, enabling comprehensive, fully automated, multi-dimensional staging and grading of periodontal disease.

## Data Availability

The original contributions presented in the study are included in the article/Supplementary Material, further inquiries can be directed to the corresponding authors.
